# Evaluating automatically generated normal tissue contours for safe use in head and neck and cervical cancer treatment planning

**DOI:** 10.1002/acm2.14338

**Published:** 2024-04-12

**Authors:** Raphael Douglas, Adenike Olanrewaju, Raymond Mumme, Lifei Zhang, Beth M. Beadle, Laurence Edward Court

**Affiliations:** ^1^ Department of Radiation Physics The University of Texas MD Anderson Cancer Center Houston Texas USA; ^2^ Department of Radiation Oncology Stanford University Stanford California USA

**Keywords:** auto‐contour evaluation

## Abstract

**Purpose:**

Volumetric‐modulated arc therapy (VMAT) is a widely accepted treatment method for head and neck (HN) and cervical cancers; however, creating contours and plan optimization for VMAT plans is a time‐consuming process. Our group has created an automated treatment planning tool, the Radiation Planning Assistant (RPA), that uses deep learning models to generate organs at risk (OARs), planning structures and automates plan optimization. This study quantitatively evaluates the quality of contours generated by the RPA tool.

**Methods:**

For patients with HN (54) and cervical (39) cancers, we retrospectively generated autoplans using the RPA. Autoplans were generated using deep‐learning and RapidPlan models developed in‐house. The autoplans were, then, applied to the original, physician‐drawn contours, which were used as a ground truth (GT) to compare with the autocontours (RPA). Using a “two one‐sided tests” (TOST) procedure, we evaluated whether the autocontour normal tissue dose was equivalent to that of the ground truth by a margin, *δ*, that we determined based on clinical judgement. We also calculated the number of plans that met established clinically accepted dosimetric criteria.

**Results:**

For HN plans, 91.8% and 91.7% of structures met dosimetric criteria for automatic and manual contours, respectively; for cervical plans, 95.6% and 95.7% of structures met dosimetric criteria for automatic and manual contours, respectively. Autocontours were equivalent to the ground truth for 71% and 75% of common DVH metrics for the HN and cervix, respectively.

**Conclusions:**

This study shows that dosimetrically equivalent normal tissue contours can be created for HN and cervical cancers using deep learning techniques. In general, differences between the contours did not affect the passing or failing of clinical dose tolerances.

## 1 INTRODUCTION

Radiotherapy is a crucial modality for treatment of head and neck (HN) and cervical cancers. Intensity‐modulated radiotherapy and volumetric‐modulated arc therapy (VMAT) are accepted treatment methods for these cancers but are subject to inter‐ and intra‐planner variability.^1^, ^2^, ^3^, ^4^ These variations can lead to poor clinical outcomes for patients treated with suboptimal plans.^3^, ^5^ Knowledge‐based planning has been shown to reduce variability,^1^, ^2^, ^4^ while also creating high‐quality treatment plans.^6^, ^7^, ^8^

The Radiation Planning Assistant (RPA) is a web‐based automated treatment planning tool being developed to generate consistent, high‐quality contours and treatment plans. The development of the RPA has been previously described.^7^, ^9^, ^10^, ^11^, ^12^, ^13^, ^14^, ^15^, ^16^, ^17^, ^18^, ^19^, ^20^, ^21^ The RPA uses deep learning and knowledge‐based planning to provide high‐quality and safe contours and treatment plans, with a goal of improving access to radiotherapy around the world.

In this study, we quantitatively evaluate the use of automatically generated organs at risk (OAR) contours for automatic plan generation. To do this, we used the RPA to generate VMAT plans for a cohort of HN and cervical cancer patients. The RPA plans were created using OAR contours generated by deep‐learning models developed in‐house. We, then, dosimetrically compared them to the original contours drawn by the clinic to assess contour quality.

## 2 METHODS

### 2.1 Patient data

For this analysis, the medical records of a cohort of 54 patients with HN cancer and 39 patients with cervical cancer were retrospectively collected from our institution and de‐identified. All patients were previously treated using VMAT. The original physician‐drawn target contours, computed tomography (CT) scans, and dose prescriptions were used in autoplan generation. Of the 54 patients with HN cancer, 8 were originally planned using two PTV dose levels. Of 39 cervical cancer plans, 26 included a boost to the gross tumor volume. Tables 1 and 2 show the patient cohort's various subsites, prescription ranges, and fraction ranges.

**TABLE 1:** Distribution of the head and neck cancer cohort by site and total dose range.

Primary site	Number of patients	Range of PTV1 dose, Gy	Range of fractionation
Oropharynx	20	60–70	30–33
Oral cavity	11	60–70	30–33
Larynx	13	60–70	30–35
Hypopharynx	3	60–70	30–33
Nasopharynx	7	60–66	30–33

**TABLE 2:** Distribution of the cervical cancer cohort by site and total dose range.

Primary site	Number of patients	Range of total dose, Gy	Range of fractionation
Pelvis	29	43.2–50	25
Para‐aortic lymph node/pelvis	10	45–50	24–28

### 2.2 RPA workflow

Plan generation in the RPA is fully automated. The only user input required is the upload of the CT images and the service request, which contains the dose prescription, determination of margins, etc. Once the CT and service request is submitted by the user, normal tissue and target contours are generated using deep‐learning models. After the contours are created, VMAT plans are generated and optimized using RapidPlan models in the Eclipse treatment planning system (TPS) (Varian Medical Systems, Palo Alto, CA). Both the contouring and RapidPlan models were developed by our group.^7^, ^18^, ^19^ After the autoplan is generated, the user can download the autocontours and plan files from the website interface to import into their own treatment planning system. The full RPA workflow^9^ and model performances^7^, ^17^, ^18^, ^19^, ^20^ used in this study have been outlined in previous publications. The plans used in this study were generated for use on a Varian 2100 machine. The HN plans consisted of three 360° coplanar treatment arcs with collimator angles of 15°, 345°, and 90°. For cervical plans, three 360° coplanar treatment arcs were used at collimator angles of 10°, 350°, and 90°. The RapidPlan models use 6 MV photon beams. The anisotropic analytical algorithm (AAA) was used to calculate dose. The autoplans have been shown to be clinically acceptable in previous studies.^7^, ^21^

### 2.3 Plan generation and data collection

For this study, we wanted to dosimetrically compare the autocontours created by the RPA to the original, physician‐drawn (manual) contours. To do this, we needed to apply the same plan across both sets of contours. First, we generated an autoplan using the RPA. We, then, imported the manual contours into our Eclipse TPS, alongside the autocontours and autoplan. This was done for each patient in our patient cohort. The reported dose to the manual contour was considered our ground truth (GT) and we used it to evaluate the reported dose to the autocontours (RPA). Dosimetric data were collected using a Python script to interface with the Eclipse Scripting API.

### 2.4 Evaluation process

In this evaluation, we used a “two one‐sided tests” (TOST) procedure^22^, ^23^, ^24^ to determine if the dose to the autocontour was equivalent to that of the ground truth by a margin, *δ*, that we determined based on clinical judgment. Below are our hypotheses:

$$H_{0}:\;M_{\mathrm{RPA}}\leq M_{\mathrm{GT}}-\delta \;\mathrm{or}\;M_{\mathrm{RPA}}\geq M_{\mathrm{GT}}+\delta$$

$$H_{1}:\;M_{\mathrm{GT}}-\delta <M_{\mathrm{RPA}}<M_{\mathrm{GT}}+\delta$$

*M*_RPA_ is the median dose to the autocontour and *M*_GT_ is the median dose for the ground truth, for a given normal structure DVH metric. For volumetric comparisons, *δ* = 5%; for dosimetric comparisons, *δ* = 3.5 Gy (or 5% of 70 Gy) for HN patients and *δ* = 2.5 Gy (or 5% of 50 Gy) for cervical patients. We did not consider the target contours in this evaluation. Using a one‐sided Mann‐Whitney *U* test, we considered the autocontour and the manual contour equivalent if both null hypotheses are rejected (i.e., *p* < 0.10) and a 90% confidence interval for *M*_RPA_–*M*_GT_ lies between (−*δ*, *δ*).

We also calculated the number of plans that met established clinically accepted dosimetric criteria. For HN plans, the criteria used is outlined in Radiation Therapy Oncology Group protocol 1016.^25^ For cervical plans, the criteria used is based on the GEC‐ESTRO EMBRACE II protocol^26^ and our own internal protocol.

## 3 RESULTS

### 3.1 Equivalence

**FIGURE 1:**
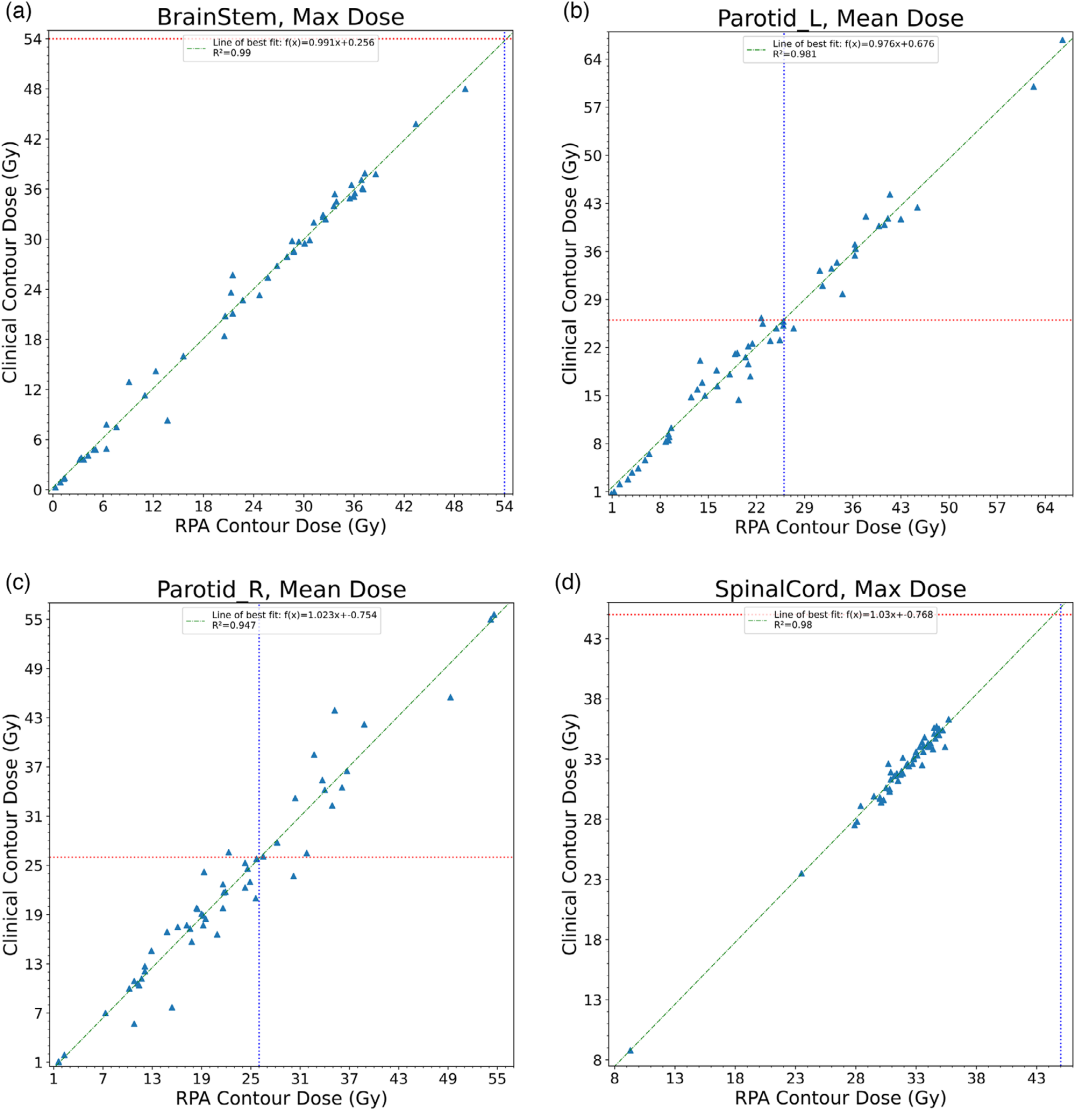
Scatterplots compare the distribution of planned dose to the normal structures for the brainstem (a), left parotid (b), right parotid (c), and spinal cord (d). The green line represents the estimated regression function. The red and blue lines represent the dosimetric constraints for a given normal structure.

**FIGURE 2:**
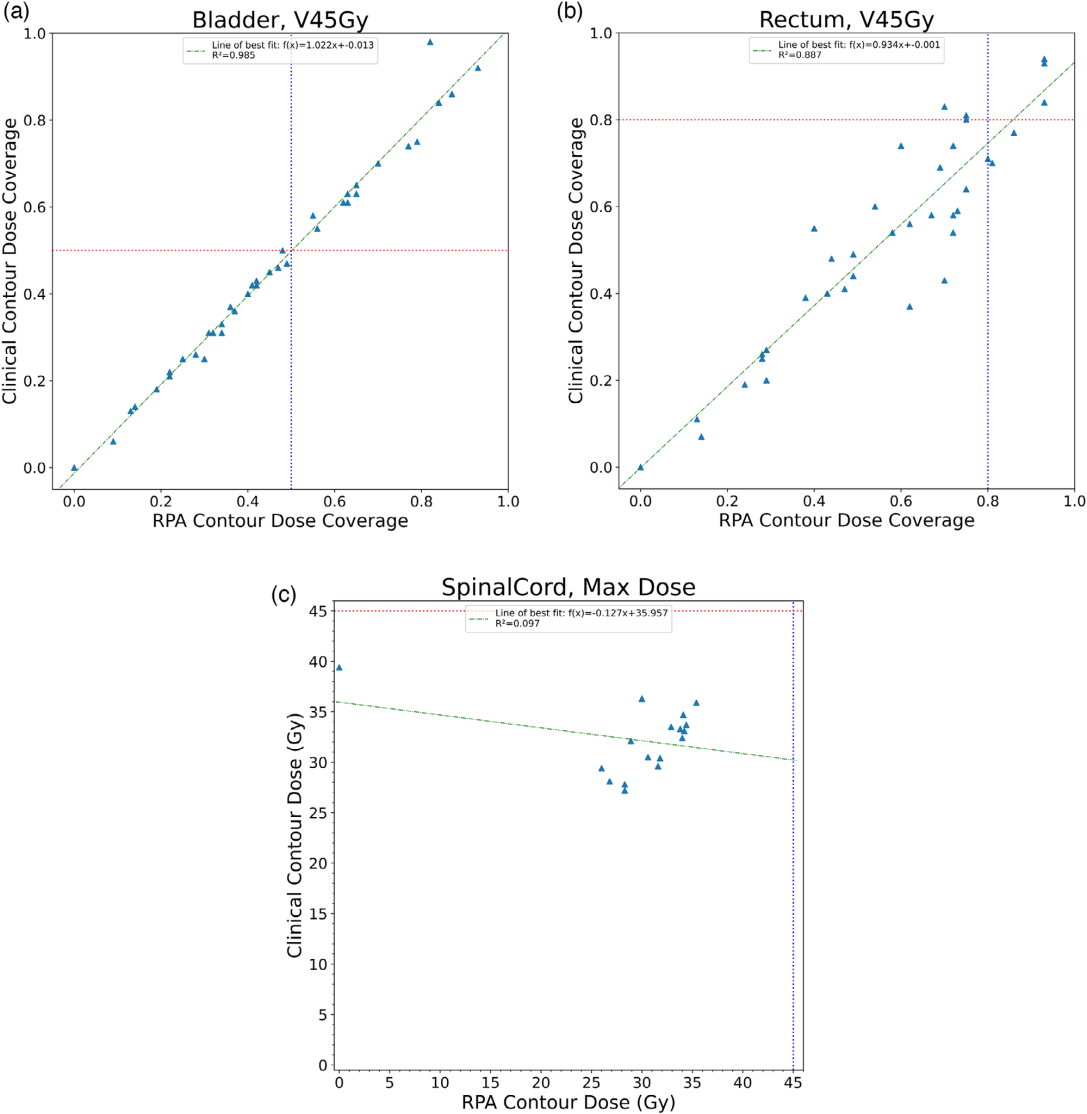
Scatterplots compare the distribution of planned dose to the normal structures for the bowel bag (a), bladder (b), and rectum (c). The green line represents the estimated regression function. The red and blue lines represent the dosimetric constraints for a given normal structure.

The autocontours were equivalent (*p* < 0.10) to the ground truth for 12 of 17 HN DVH metrics and 15 of 20 cervical DVH metrics. Tables 3 and 4 show the confidence interval and *p*‐values. Some OARs were not contoured for every patient. So, the number of data points used in the test for a given structure was included to help provide context. *P*_0_ represents the *p*‐value result of testing null hypothesis *M*_RPA_ ≤ *M*_GT_‐*δ*; while. *P*_1_ represents the *p*‐value result of testing null hypothesis *M*_RPA_ ≤ *M*_GT_‐*δ*. Both *P*_0_ and *P*_1_ need to be less than 0.10 to show equivalence. Figure 1 shows the distribution of planned dose to the brainstem, bilateral parotid glands, and spinal cord for the HN cancer cases, and Figure 2 shows the distribution for the bladder, bowel bag, and rectum for the cervical cancer cases.

**TABLE 3:** 90% confidence intervals (CIs) and *p*‐values of the TOST for the HN cancer cases.

Structure, metric	90% CI	*p*_0_‐value	*p*_1_‐value	Number of data points
Brain, max dose	(−3.1, 4.2)	0.088	0.134	54
BrainStem, max dose	(−2.6, 2.5)	0.047	0.040	54
Chiasm, max dose	(−15.9, 16.5)	0.583	0.201	5
Left cochlea, max dose	(−0.6, 1.0)	<0.001	<0.001	54
Right cochlea, max dose	(−0.6, 0.7)	<0.001	<0.001	54
Left eye, max dose	(−0.5, 0.2)	<0.001	<0.001	38
Right eye, max dose	(−0.5, 0.2)	<0.001	<0.001	38
Left Lens, max dose	(−0.2, 0.2)	<0.001	<0.001	53
Right lens, max dose	(−0.2, 0.2)	<0.001	<0.001	53
Mandible, max dose	(−1.1, 0.9)	0.012	0.009	54
Left OpticNrv, max dose	(−10.1, 7.1)	0.273	0.236	10
Right OpticNrv, max dose	(−8.4, 6.2)	0.285	0.192	10
Left parotid, mean dose	(−3.6, 3.0)	0.109	0.073	54
Right parotid, mean dose	(−2.2, 2.8)	0.038	0.065	54
SpinalCord, max dose	(−0.8, 0.4)	<0.001	<0.001	54
zBrainStem_05, max dose^a^	(−3.1, 3.4)	0.083	0.093	52
zSpinalCord_05, max dose^a^	(−0.8, 0.9)	<0.001	<0.001	54

Abbreviations: HN, head and neck; TOST, two one‐sided tests.

^a^ Structure with a 5‐mm margin.

**TABLE 4:** 90% confidence intervals (CIs) and *p*‐values of the TOST for the cervical cancer cases.

Structure, metric	90% CI	*p*_0_‐value	*p*_1_‐value	Number of data points
Bag_Bowel, V40 Gy	(0.04, 0.08)	<0.001	0.753	36
Bladder, V45 Gy	(−0.07, 0.08)	0.169	0.209	39
Femoral heads, max dose	(−1.8, 0.2)	0.024	0.001	34
Femoral heads, V40 Gy	(0, 0)	<0.001	<0.001	34
Femoral heads, V45 Gy	(0, 0)	<0.001	<0.001	34
Femur_Head_L, max dose	(−2.7, 0.3)	0.129	0.002	27
Femur_Head_L, V40 Gy	(0, 0)	<0.001	<0.001	27
Femur_Head_L, V45 Gy	(0, 0)	<0.001	<0.001	27
Femur_Head_R, max dose	(−2.0, 0.4)	0.061	0.004	27
Femur_Head_R, V40 Gy	(0, 0)	<0.001	<0.001	27
Femur_Head_R, V45 Gy	(0, 0)	<0.001	<0.001	27
Kidney_L, mean dose	(−1.8, 1.6)	0.053	0.065	15
Kidney_L, V15 Gy	(−0.03, 0.02)	0.058	0.051	15
Kidney_L, V20 Gy	(0, 0)	0.002	0.001	15
Kidney_R, mean dose	(−1.3, 1.3)	0.032	0.045	15
Kidney_R, V15 Gy	(−0.04, 0.04)	0.073	0.076	15
Kidney_R, V20 Gy	(0, 0.01)	0.008	0.006	15
Liver, V35 Gy	(0, 0.01)	0.004	0.004	5
Rectum, V45 Gy	(−0.04, 0.12)	0.076	0.409	39
SpinalCord, max dose	(−2.5, 0.6)	0.107	0.002	17

Abbreviation: TOST, two one‐sided tests.

### 3.2 Dosimetric criteria

The plans met dosimetric criteria for 91.8% and 91.7% of all HN structures for generated and manual contours, respectively. For cervical plans, 95.6% and 95.7% of structures met dosimetric criteria for automatically generated and manual contours, respectively. Tables 5 and 6 list the DVH metrics evaluated for each site and the number of plans that met criteria.

**TABLE 5:** Number of HN autocontours (RPA) and manual contours (GT) that met clinical dose criteria.

Structure	Metric, criteria	RPA	GT	Number of data points
Brain	Max dose < 70 Gy	54	54	54
BrainStem	Max dose < 54 Gy	54	54	54
Chiasm	Max dose < 54 Gy	5	4	5
Left cochlea	Max dose < 35 Gy	50	51	54
Right cochlea	Max dose < 35 Gy	50	50	54
Left eye	Max dose < 54 Gy	37	38	38
Right eye	Max dose < 54 Gy	37	37	38
Left kens	Max dose < 7 Gy	50	50	53
Right kens	Max dose < 7 Gy	50	50	53
Mandible	Max dose < 70 Gy	45	45	54
Left OpticNrv	Max dose < 54 Gy	10	9	10
Right OpticNrv	Max dose < 54 Gy	10	9	10
Left parotid	Mean dose < 26 Gy	36	36	54
Right parotid	Mean dose < 26 Gy	38	38	54
SpinalCord	Max dose < 45 Gy	54	54	54
zBrainStem_05^a^	Max dose < 54 Gy	50	50	52
zSpinalCord_05^a^	Max dose < 50 Gy	54	54	54

Abbreviations: GT, Ground Truth; HN, head and neck; RPA, Radiation Planning Assistant.

^a^ Structure with a 5‐mm margin.

**TABLE 6:** Number of cervical autocontours (RPA) and manual contours (GT) that met clinical dose criteria.

Structure	Metric, criteria	RPA	GT	Number of data points
Bag_Bowel	V40 Gy < 30%	33	34	36
Bladder	V45 Gy < 50%	25	25	39
Femoral heads	Max dose < 50 Gy	34	34	34
Femoral heads	V40 Gy < 15%	34	34	34
Femoral heads	V45 Gy < 60%	34	34	34
Left Femur_Head	Max dose < 50 Gy	27	27	27
Left Femur_Head	V40 Gy < 15%	27	27	27
Left Femur_Head	V45 Gy < 60%	27	27	27
Right Femur_Head	Max dose < 50 Gy	27	27	27
Right Femur_Head	V40 Gy < 15%	27	27	27
Right Femur_Head	V45 Gy < 60%	27	27	27
Left kidney	Mean dose < 18 Gy	15	15	15
Left kidney	V15 Gy < 50%	15	15	15
Left kidney	V20 Gy < 33%	15	15	15
Right kidney	Mean dose < 18 Gy	15	15	15
Right kidney	V15 Gy < 50%	15	15	15
Right kidney	V20 Gy < 33%	15	15	15
Liver	V35 Gy < 50%	5	5	5
Rectum	V45 Gy < 80%	34	34	39
SpinalCord	Max dose < 45 Gy	17	17	17

Abbreviations: GT, Ground Truth; RPA, Radiation Planning Assistant.

## 4 DISCUSSION

Overall, this analysis demonstrates that autocontours were equivalent to the ground truth for 71% and 75% of common DVH metrics for the HN and cervix, respectively. For DVH metrics that did not meet our equivalence criteria, the autocontours tended to result in a higher reported dose than the ground truth. This may be a result of the autocontours being somewhat more generous than the manual contours, meaning they are more likely to report a higher dose. This finding can be seen in the bowel bag and rectum for cervical cancer and the brain for HN cancer, where the upper limit of the confidence interval was greater than the *δ* and the lower limit was within our margin. For the left parotid (HN), left femoral head (cervical) and spinal cord (cervical), the opposite was true; the autocontours tended to report less dose to these structures than the ground truth contours. Some of the DVH metrics were not able to confirm equivalence due to a lack of data (as not all structures are manually contoured for all patients), in particular, the optic chiasm (5 plans), and both optic nerves (10 plans each). This is also true for the bladder, despite having significantly more data points (39) than the optic chiasm and nerves. We were, however, able to confirm equivalence for the liver contour (5 plans).

In general, even for structures that did not demonstrate equivalence, the autocontours were just as safe as the manual contours for planning for a majority of the DVH metrics. For almost all DVH metrics, the same number of autocontours and ground truth contours met dosimetric criteria. The exceptions are the optic chiasm, both optic nerves, left cochlea, and left eye, which there is a difference of one plan between the two planning sets. Although preliminary, this indicates that effort should be spent reviewing all HN and cervical structures, especially when they are near tolerance, as they will tend to be in a dose gradient, so contouring errors will be particularly impactful.

This study has some limitations. Many of the patient data in our dataset were missing some clinical contours that we used in our evaluation. Depending on the treatment area, these contours were not critical for the treatment of these patients and were not delineated; however, we would need to increase the number of patients in our dataset or curate the patient structure files prior to reproducing this study. This study focused on patients from our own institution and did not evaluate the effect of anatomical variations, patient characteristics, clinical scenarios, and other factors on contour quality. In future work, we would need to widen the scope of our study by diversifying our cohort, using patients from other institutions and take the aforementioned factors into account. This would allow us to evaluate the generalizability of our models. In general, changes to CT resolution and acquisition on different scanners can affect the accuracy of autocontouring models. However, in a publication by Huang, et al.^27^ showed that deep‐learning contouring models a particular robust to pixel size and slice thicknesses >3 mm. Since the RPA does not allow for CT images with slice thickness above 3 mm, CT image quality was not considered.

In this study, we showed that many, but not all, structures are dosimetrically equivalent when comparing automatically generated and manual structures. Differences in contouring did not, however, generally affect whether the structures passed or failed clinical tolerances. Although rare, there were situations where the reported dose indicated that the autoplan passed clinical criteria, but the auto‐generated contour did not meet equivalence, thus showing that careful contour review is still important.

## AUTHOR CONTRIBUTIONS

Laurence Court, Adenike Olanrewaju, and Lifei Zhang conceived and designed this experiment. Beth Beadle provided clinical guidance for the model used in the experiment. Raymond Mumme and Raphael Douglas carried out the experiment. Raphael Douglas performed the data collection and analysis of the data. Laurence Court supervised the findings in this work. Raphael Douglas wrote the initial draft of the manuscript with support of Laurence Court; all authors contributed to the final manuscript.

## CONFLICT OF INTEREST STATEMENT

The authors declare no conflicts of interest.
